# Supplementary material for: Development of Scaffolds with Adjusted Stiffness for Mimicking Disease-Related Alterations of Liver Rigidity
Source: J Funct Biomater. 2020 Mar 14;11(1):17. doi: 10.3390/jfb11010017 (PMC7151584; doi:10.3390/jfb11010017)
Supplement: Supplementary file 1 [file jfb-11-00017-s001.pdf]

materials

Supplementary Materials: Development of Scaffolds with Adjusted Stiffness for Mimicking Disease-Related Alterations of Liver Rigidity

Marc Ruoß [1,*], Silas Rebholz [1], Marina Weimer [1,2], Carl Grom-Baumgarten [1], Kiriaki Athanasopulu [2], Ralf Kemkemer [2], Hanno Käß [3], Sabrina Ehnert [1] and Andreas K. Nussler [1]

Table S1. Concentrations of cryogel components which were tested during the development of the scaffolds.

Scaffold Component	Final Concentration Within the Cryogel
ddH_2O	adapted to the other scaffold components
2-HEMA 98%	1%–30%
BAA 2%	ratio 1:1 – 1:6 adapted to the HEMA concentration
Gelatin (300 g/L)	0%–40%
BSA (100 g/L)	0%–60%
Collagen (3.5 g/L)	0%–40%
TEMED	2%
APS 10%	0.2%
Glutaraldehyde 25%	0%–2.5%

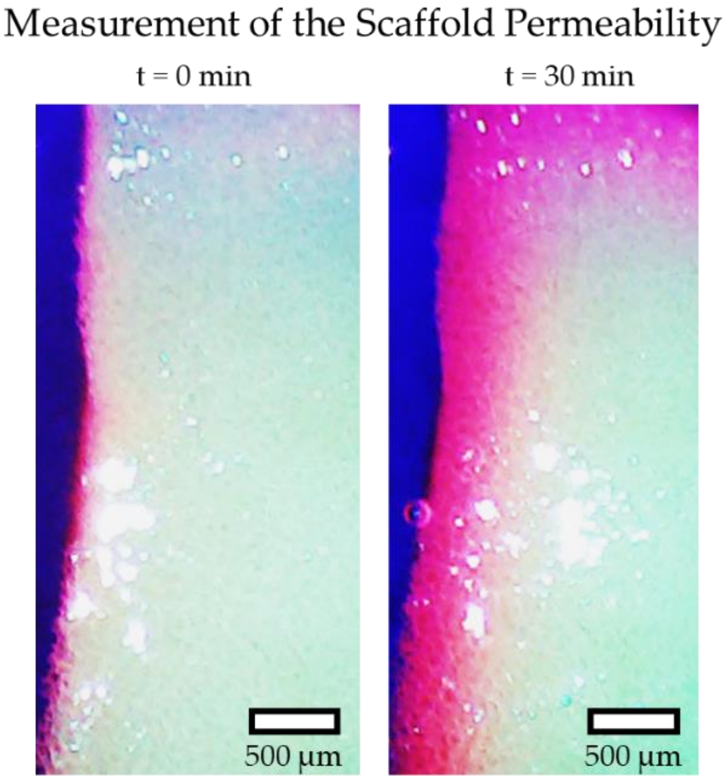

Figure S1. The diffusion rate of the red-colored SRB solution into the scaffold was used to determinate the permeability of the scaffolds. The figure shows representative images of the cross-section of the healthy liver scaffold. These images were used for the analysis of the scaffold permeability.
